# A qualitative study on the views of experts on the social impact of the high-priced orphan drug nusinersen

**DOI:** 10.1016/j.rcsop.2023.100227

**Published:** 2023-01-20

**Authors:** Sara Rosenberg, Björn Södergård, Jessica M. Rosenholm, Jussi-Pekka Rauha

**Affiliations:** a Pharmaceutical Sciences Laboratory, Faculty of Science and Engineering, Åbo Akademi University, 20520 Turku, Finland; b Hospital Pharmacy, Vaasa Central Hospital, 65130 Vaasa, Finland

**Keywords:** Nusinersen, Orphan drug, Social impact, Medical costs, Qualitative research

## Abstract

**Background:**

Escalating medical costs due to the increasing occurrence of high-priced orphan drugs is a topic discussed in the media and specialist literature. However, there is no study investigating the social impact of such drugs through the views of experts.

**Objectives:**

The aim was to demonstrate the social impact of the orphan drug nusinersen based on the views of experts within the community.

**Methods:**

The study was conducted using two methods for data collection: a media analysis and qualitative semi-structured interviews. In the media analysis, expert comments on nusinersen were extracted from the Finnish media. Interviews were conducted with experts from the fields of pharmacy, medicine, politics/academia, law/economics, hospital management and patient organisations from different parts of Finland, who encountered nusinersen in their profession. Participants were recruited through purposive and snowball sampling. Interviews were audio-recorded, transcribed verbatim, and the overall data were analysed thematically.

**Results:**

Twenty-nine media references were collected, and 16 interviews conducted. Three main themes were identified: ethical aspects, financial aspects, and call for new strategies. Expert views were divided between the ethical and financial aspects of nusinersen. These existed alongside each other, showing that different attitudes and values compete with each other, and may be classified in different ways depending on the situation. However, the discussion quickly evolved into a call for new strategies in order to find solutions to issues concerning orphan drugs and the social impact created as a result.

**Conclusions:**

This study reveals the social impact of nusinersen thus far within the community and it appears somewhat different when seen from the perspectives of patients and decision-makers. Even though impact has been created, such as the establishment of a disease-specific patient organisation, other issues still require further research. Among these are the potential establishment of international collaboration forums for price negotiations with pharmaceutical companies.

## 1 Introduction

Research regarding rare diseases and the development of orphan drugs has progressed in the past few decades, partly due to the sequencing of the human genome and the resulting faster and cheaper ability to associate between genetic mutation and disease,^1^ but also as a result of a benign development in legislation in both the US and Europe.^2^^,^^3^ The price of orphan drugs is generally high due to factors like the unavailability of alternative options and the timeframes of marketing exclusivity.^4^ The price also increases further when these drugs are used for treating a rare disease with a very low prevalence such as spinal muscular atrophy (SMA).

Nusinersen (trade name Spinraza) was the first medical treatment available for the severely debilitating hereditary neurodegenerative disorder SMA.^5^^,^^6^ It was approved for use in the European Union (EU) in May 2017.^7^ The drug is classified as an orphan drug^8^ and is one of the most expensive medical treatments in the world,^9^ with a list price in Finland of 500,000 € per patient for the first year of treatment and 250,000 € per patient for subsequent years.^10^ The price of the drug is not expected to go down for at least as long as its patent is valid.^11^ Currently in Finland, approximately 40 out of 100 SMA-patients are treated with nusinersen, and 4–5 children with SMA are born every year.^12^

During the spring of 2018 the Council for Choices in Health Care in Finland (COHERE Finland) included the drug in the services financed by public funds, after an agreement regarding a price reduction between university hospitals and the manufacturer had been reached.^13^^,^^14^ The agreement, however, remains classified. In 2018–2022, COHERE Finland recommended the use of nusinersen for SMA-patients under 18 years of age, who had been diagnosed before the age of 2, who were symptomatic before 20 months of age, and who were not in need of continuous ventilation and did not have another medical impediment for treatment.^13^ Recently, the recommendation has been updated.^15^ Patients receiving the treatment are continuously followed up and the recommendation is to continue the treatment for as long as the patient presents verified clinical benefits.

Although nusinersen is used in most parts of Europe, due to different pricing and reimbursement policies the availability of the drug is also different in the different European countries.^16^ SMA patient organisations throughout Europe have joined forces to make effective therapies available to their patient groups.^17^

Currently, high-priced orphan drugs of this kind are becoming increasingly common. For example, two alternative drug treatments for SMA have recently been approved in the EU. Zolgensma was approved in May 2020^18^ and Evrysdi in March 2021^19^ with price tags of around $2.1 million as a one-time therapy and a maximum annual cost of $340,000 per patient, respectively (before possible country-specific discounts).^9^^,^^20^ Zolgensma was included in the services financed by public funds in Finland in November 2021, however, with a demand for a substantial price reduction.^21^ Evrysdi is currently not included and is thus unavailable to Finnish SMA-patients. As with many orphan drugs, traditional cost-effectiveness analyses are difficult to conduct due to the limited amount of available clinical data at the time of marketing authorisation and the cost-effectiveness is mainly estimated by extrapolation of existing data.^22^, ^23^, ^24^ This raises questions concerning the equality in care and financing in relation to high-priced treatments. Expensive orphan drugs such as nusinersen have thus received extensive media attention during the past two decades.

By definition,^25^ social impact is the net effect of an activity on a community and the well-being of individuals and families. It is a significant or positive change that addresses social injustice or challenges, and in best case scenarios solves them.^26^ These changes may be achieved through conscious activities in the operations and administrations of organisations within the public, private and fourth sectors. The social impact concept was introduced in the early 1970s by social entrepreneur Bill Drayton. Social impact can be created through any of the 17 Social Development Goals (SDGs) set by the United Nations (UN) as a way to work towards making a significant impact in the world and creating opportunities otherwise unavailable to minorities or the underprivileged.^27^ Out of these goals, nr. 3 is *Good Health and Well-being* and nr. 10 is *Reduced Inequality*. Therefore, the social impact concept may well be applied within the context of high-priced orphan drugs like nusinersen, in searching for a sustainable and beneficial systems change, as such drugs are accompanied by diverse implications on communities as well as individuals and families.

Ethical implications in relation to orphan drugs have been reviewed over the years.^23^^,^^28^, ^29^, ^30^, ^31^ Also, other implications of nusinersen have been previously studied, thus far mainly from financial^22^, ^23^, ^24^ and patient perspectives.^32^, ^33^, ^34^, ^35^ This study, investigating the social impact of nusinersen as seen from a novel, expert perspective, strives to provide valuable insights, offering additional awareness and guidance for policymakers, regulatory agencies and industry, and identifying areas needing further investigation and policy development.

## 2 Aim of the study

The aim of this study was to demonstrate the social impact of a high-priced orphan drug, nusinersen, via perceptions, thoughts and attitudes of involved experts within the community.

## 3 Methods

### 3.1 Study design and recruitment

The study was commenced by a media analysis as a method of data collection,^36^ to search for expert comments on nusinersen and high-priced orphan drugs in the Finnish media. By definition,^37^ media analysis is the study of what is said on a given subject in a given place at a given time within the media. It thereby presented a suitable gateway for the study at hand, as there were much data to obtain from both regular and specialist media in Finland, concomitantly forming a relevant literature search on the subject matter.

Semi-structured qualitative interviews were thereafter conducted with experts in different areas of expertise from around Finland who through their profession in various ways encounter nusinersen and other orphan drugs. These experts covered the fields of pharmacy (hospital as well as community), medicine, politics and academia, law and economics, hospital management and patient organisations. Patient organisations were included within the group of experts as they hold valuable information concerning the patient group. This type of qualitative interview was, in combination with the media analysis, considered to be a suitable method for collecting data when attempting to elucidate the thoughts and views of experts on this specific subject matter and identifying themes related to it.^38^

Participants were recruited primarily through purposive sampling by contacting experts within Finnish hospitals, community pharmacies and patient organisations, and subsequently, to a smaller extent, through snowball sampling as a few interview participants/decliners spontaneously suggested other potential participants.^39^ The first author (SR) invited participants by phone or email to be interviewed individually. Contact details of the participants were retrieved from the homepages of their respective organisations. Before the interviews, the eligibility of the participants had been evaluated by two members of the research team (SR, JPR). Eligibility criteria were that the experts should encounter high-priced orphan drugs like nusinersen in their profession or by representing patient organisations. Experts that had commented on the subject matter in media were excluded from the pool of potential interview participants in order to broaden the scope of the results. Also, the researchers wished to include pharmacists, a group which was somewhat underrepresented in the media material. All participants were informed of the purpose of the study, that participation was voluntary and that they were free to withdraw from the study at any time of the interview process (see Appendix A, Supplementary data). No compensation for participation was provided.

### 3.2 Data collection

The collection of data was commenced by the first author (SR) by reviewing articles on the subject matter in Finnish regular and specialist media published between May 2017 and May 2018 (see Appendix D, Supplementary data), excluding social media and TV. The search tool Google and search terms *nusinersen* and *Spinraza* were used for the keyword search. This was followed by identification and extraction of expert comments found within the material (SR). All articles that were found using the search tool and -terms indicated and included expert comments on nusinersen and high-priced orphan drugs were included. The outset of the period was determined as the date when nusinersen was authorised for use in the EU, which highly increased its visibility in the media.

The interview guide was constructed by two of the researchers (SR, JPR), based on the preliminary findings of the media analysis and the aim of the study. The content of the interview guide was the same for all participants apart from the initial question, which was tailored to specifically address the different fields of expertise (see Appendix B, Supplementary data). The interview guide was piloted by interviewing an expert within the field of law and economics. The pilot interview did not result in any revisions to the interview guide and the findings from this interview were not included in the study. During the interview recruitment process, the first author (SR) found some expert groups harder to get in touch with than others (patient organisation vs pharmacy), leading to two additional interviews being made at a later point in time. Two participants, from the expert groups hospital management and community pharmacy, declined the invitation to participate, both however suggesting another participant from their respective fields of expertise. Four potential participants did not respond at all.

The interviews were conducted in two stages, first from October 2018 to January 2019 and also in May 2021 due to delays in the recruitment process as explained above. The interviews were conducted face-to-face or by telephone by the first author (SR) and lasted from 20 to 40 min. The interviewer had no established relationship with any participant. Face-to-face interviews were conducted at a neutral and undisturbed location chosen by the participant in order to make the participant feel as comfortable as possible (e.g., the workplace of participant or a quiet corner in a cafeteria). All participants provided their written informed consent prior to the interview (see Appendix C, Supplementary data). The interviews were conducted in Finnish or Swedish, depending on the mother tongue of the participant, an issue not presenting a problem as the first author (SR) is bilingual. Participants were given the opportunity to speak at length about their views on the subject matter, interviews were audio-recorded in order to give the researcher an opportunity to react to non-verbal messages and take field notes during the interviews. The interviews were transcribed verbatim by the first author (SR). All data were handled confidentially: anonymised, saved solely in the first authors (SR) personal computer and will be deleted after the finalisation of the study. Transcripts were not returned to participants for review. However, an attempt was made to sort out all noted miscommunications and incomplete answers during the interview by verifying possible uncertainties directly from the participant by probing questions. The participants were also given the opportunity to read and comment on the finalised study prior to publication. Those (14 out of 16 participants) who commented on the study were satisfied with it and there were no requests to revise the content.

### 3.3 Data analysis

The data analysis was initiated at an early stage of the study as articles from the media regarding nusinersen and high-priced orphan drugs were read and re-read by the first author (SR), manually extracting significant quotes by experts whilst making a preliminary inductive analysis using a semantically descriptive approach. The articles were analysed as qualitative data using a thematic analysis scheme reported by Braun and Clarke,^40^ as this method of analysis is flexible and therefore suitable for analysing different types of data.

All interviews were manually transcribed and the correctness of the transcripts was double checked by the first author (SR). This was done as quickly as possible, preferably on the same day, or the day after the interview at the latest. The analysis of the interviews was initiated after the first interview. The transcripts were read and re-read as a first step of the analysis. The first author (SR) analysed the transcribed data, again using the thematic analysis scheme by Braun and Clarke.^40^ After manual coding and categorisation of initial codes to themes, a second member of the research team (JPR) independently read an arbitrary sample of the interviews in order to confirm the themes as being reflective of the data content through a discussion with the first author (SR). At the final stages of the data analysis, the results of the media analysis were read anew (SR, JPR) and, through further refinement and discussions, combined with the interview results (SR, JPR, BS). At this point, the results indicated saturation according to the Francis method,^41^ giving the sample size presented in Table 1. An initial analysis sample of ten and a stopping criterion of three for the interviews was agreed upon a priori by the authors. The stopping criterion was tested after each consecutive interview until the last three interviews were without additional material (i.e., interviews 14, 15 and 16). Finally, combining the media- and interview data verified that the sample size with respect to the six different areas of expertise was adequate.

**Table 1:** Expert codes and the number of representatives of the different fields of expertise in the media and interviews.

Area of expertise	Expert code⁎	Interviews	Media extracts⁎⁎, ⁎⁎⁎	Total
Pharmacy	Ph	5	2	7
Medicine	Me	2	5	7
Politics & academia	Pa	2	4	6
Law & economics	Le	2	4	6
Hospital management	Hm	2	3	5
Patient organisation	Po	3	4	7

⁎ Codes are used in results in referring to example quotes given and by which field of expertise: I Po1 = quote from interview response by representative nr.1 of patient organisation.

⁎⁎ The number of experts in media extracts (22) differs from the total number of media references (29) as some experts appeared in several articles.

⁎⁎⁎ See Appendix D. Supplementary data for media references and a source/chronological distribution thereof.

### 3.4 Ethical considerations

Permission to conduct this study was granted by the Central Hospital in Vaasa, Finland (VKS-2021-45-JYL). According to the ethical committee of Southwest Finland at the Turku University Hospital, no ethical approval was necessary as this type of study is exempted according to the Finnish Medical Research Act.^42^

## 4 Results

The number of media references containing expert comments from various Finnish newspapers, journals and relevant home pages landed on 29 articles. The number of interview participants, on the other hand, was 16. Representatives of the six different fields of expertise were fairly evenly distributed in the summarised results (Table 1). The characteristics of the interview participants and the duration of the interviews are presented in Table 2.

**Table 2:** Characteristics of the interview participants and the duration of the interviews.

	Pharmacy (*n* = 5)	Medicine (*n* = 2)	Politics & (n = 2) academia	Law & (n = 2) economics	Hospital (n = 2) management	Patient (*n* = 3) organisation
Gender						
Male n (%)	2 (40.0%)	2 (100.0%)	2 (100.0%)	1 (50.0%)	1 (50.0%)	0 (0.0%)
Female n (%)	3 (60.0%)	0 (0.0%)	0 (0.0%)	1 (50.0%)	1 (50.0%)	3 (100.0%)
Median age in years	51.6	54.0	53.0	50.5	56.5	54.3
Median duration	24 min 9 s	16 min 0 s	22 min 50 s	14 min 28 s	21 min 5 s	33 min 50 s
Expert role(s)	Chief pharmacist	Specialist (neurology) Specialist (pharmacology)	Politician	Economist Lawyer	Medical director Head nurse	–
Work sites (n)	Community phar. (3) Hospital phar. (2)	Hospital	Local politics (1) National politics (1)	Hospital	Hospital	Elected official (1) Member (2)

**Fig. 1:**
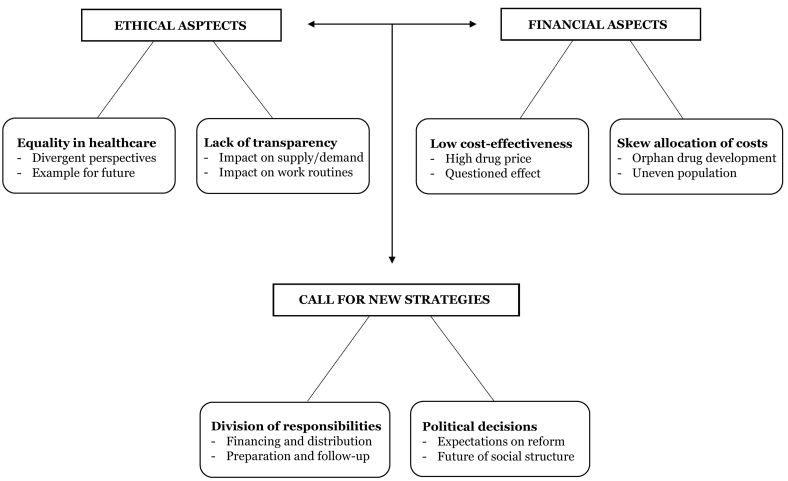
Thematic map showing the two main themes giving rise to a third main theme.

The thematic map shown in Fig. 1 forms the overall result of the analysis. The analysis resulted in three main themes with two sub-themes, respectively: 1. Ethical aspects with the sub-themes equality in healthcare and lack of transparency, 2. Financial aspects with the sub-themes low cost-effectiveness and skew allocation of costs, and 3. Call for new strategies with the sub-themes division of responsibilities and political decisions. As depicted in the thematic map, a clear connection was found between the first two main themes, ethical and financial aspects, together leading to the third main theme, call for new strategies.

### 4.1 Ethical aspects

#### 4.1.1 Equality in healthcare

A vast part of opinions concerning nusinersen landed within the frames of ethical aspects and reflections on prioritisation and equality in healthcare. The unmeditated attitude towards the drug was positive from a patient as well as drug development point of view. However, the patient organisation group experienced the Finnish priority setting to be unfair. They advocated that the drug should be reimbursed for patients having a benefit from it and pointed out that not all even want to try it, *“EVERYONE should have the same opportunity since it is not known exactly how it works, or for who it works”* (I Po3); *“But I still think that the drug should be made available to all patients that want it since not everyone wants it”* (I Po2). In the media, the patient organisation group also raised a request for open, face to face communication between patients and decision-makers working with prioritisation. On the other hand, the majority of interview participants from other expert groups expressed their trust in the system and found the priority setting to be fair. In this context, the pressure, difficultness and aloneness of decision-making was emphasised, *“… according to what we have discussed with physicians here, it is equally difficult this thing* [prioritisation]*. So, who has the courage to draw that line then?”* (I Ph5).

Within the equality discussion, it was evident that the patients were seen to have taken a more active role in demanding specific efforts in their care. They were seen to be willing to fight to receive care despite the financial consequences for society, *“If the healthcare districts refused to buy the drug because of its price, the patients would take the decision to the ombudsman*” (M Me5); *“… then a somewhat different behaviour has emerged in hospitals. There are people coming there who are declarative”* (I Pa1). The results also implied that the patient group used media to potentially affect decision-makers regarding nusinersen, *“I have been a bit worried when following this discussion* [featuring patients/families in media] *… this affects the thoughts of politicians and policymakers really a lot, writing this way, addressing feelings”* (I Ph3). Furthermore, a discussion on the role and influential power of patient organisations was identified. In the interviews, the representatives of patient organisations pointed out the fact that disease specific patient organisations, like that for SMA, have had to be founded for the sake of efficient lobbying which was perceived as having become more difficult over the years.

It was also believed that having to prioritise would cause animosity, firstly between members of the of SMA community and secondly between patient groups with different diagnoses,*”… at some point there will be two patients, between whom there is a minimal difference, and if one has worse ascriptions as to when the diagnosis was set you know, then it might be a matter of a month … even though in reality everyone knew that he has this disease”* (I Po1); *“Then we return to the question of equality: Why should common diseases be such, that concerning them the drug can't cost a lot? Why can it only cost much when it is a drug for an orphan disease?”* (M Me1). Experts stated that resources are limited and that we have the medicinal means to do more than we can afford to regardless of what the law has to say on the matter, *“Probably we will in the years ahead have more new products taken into use than before. We have been afraid of this for several years”* (M Hm3). In this context, statements were also made regarding the necessity of priority discussions concerning the use of new orphan drugs.

The role and importance of the prioritisation process of nusinersen was raised, as the drug is one of the first in its price bracket to have been taken into use in Finland, *“Yes, then if the volumes of such drugs increase, then the call for guidelines grows bigger of course”* (I Le2). Concerning the issue on the pharmaceutical company theoretically being able to sell the drug at a different price to different hospitals in the country, thereby potentially affecting regional equality in patient care, was seen as problematic by a majority of experts, further adding to the call for national guidelines. Some interview participants, despite recognising the issue, did not believe it to be significant as solutions are becoming available, *“This procurement concerning these high-priced drugs, a national cooperation is discussed and is to some extent implemented already even though it is in its early stages”* (I Me2). In comparisons on drug availability on an international level between government agency- and private health insurance-based reimbursement systems, the interview participants thought it beneficial that the Finnish system is in accordance with those of other Nordic countries, *“I think that we should act like the other Nordic countries, as our healthcare systems are quite alike in other aspects as well”* (I Hm1).

#### 4.1.2 Lack of transparency

When the lack of transparency between pharmaceutical companies and healthcare districts versus the public concerning price negotiations was discussed, the majority understood the necessity to follow the same protocol as seen in other countries. It was speculated that not accepting to keep the price negotiations and the agreed drug price a secret would introduce a risk that the buyer (here Finland) would be left without the drug, *“We might not get all the medicines that we would otherwise. If we have different rules than the rest of Europe, then it's not going to work. We're such a small country that it's impossible”* (I Ph4). In the media material, it was stated that secret contracts are a way to protect the price levels in the US where higher prices for drugs are generally paid as compared to the European and other markets.

The lack of transparency was found to affect the work routines of some expert groups within the study. The hospital pharmacist group addressed the anxiety experienced by physicians and hospital pharmacists due to the risk of revealing the drug price as a result of human error, *“A clear discount percentage would be the best alternative, and that one could openly talk about it … that would be the clearest system for us”* (I Ph5). These two expert groups also addressed the workload due to healthcare districts separately negotiating with the pharmaceutical companies.

### 4.2 Financial aspects

#### 4.2.1 Low cost-effectiveness

Another frequently addressed issue in the results of the current study was the financial aspects of high-priced orphan drugs and more specifically their low cost-effectiveness. The list price of nusinersen was seen as extremely high. The price was, however, put in relation to matters like the cost of prolonged intensive care and nusinersen being the only available option on the market at the time. According to many experts from different groups, however, the price was not seen as crucial in deciding on its use, *“… the price of an orphan drug cannot be the only criteria as to why the treatment is not part of the Finnish healthcare service system”* (M Le3). The unwillingness of the pharmaceutical company to lower the price of the drug was questioned, both in the light of this possibly increasing sales by increasing the number of patients treated and in commenting on the companies´ pursuit of maximum earnings, *“It is a horribly high price. … then, when it has been calculated more precisely, there is after all high coverages on the way for the pharmaceutical company”* (I Ph5). The orphan status of drugs like nusinersen and its impact on the drug price were concomitantly discussed, as experts asked if there is no ceiling to the price of such drugs, all the while expressing a concern that future orphan drugs might be even more expensive.

Overall, discussions concerning the drug price rapidly shifted to reflections on its effect. A frequently encountered notion was that the high drug price also demands a good treatment effect, *“If the money were to be taken from other parts of healthcare, these savings would produce much more damage to health than nusinersen produces benefits”* (M Pa2). The need for follow-up and potentially discontinuing treatment in cases of insufficient benefits were acknowledged as relevant aspects in connection to drug efficacy. However, as a counterweight, comments on slowing down or stopping the disease progression were also found, *“… if it turns out to actually have an effect, then this human being can probably function in society, and pay taxes and work. In that way she can pay back anyway”* (I Hm1); *“In a progressive disease, a stopped progression alone would be like winning the lottery for the patient”* (M Po3).

#### 4.2.2 Skew allocation of costs

The orphan status and regulations surrounding this, affecting the research and development (R&D) process and patents of high-priced orphan drugs like nusinersen, together with a small and unevenly distributed patient population, were clearly seen as relevant issues within the study findings. These appertain to the financial aspects as they result in a distinctive allocation of costs. An understanding of the pharmaceutical company's necessity to earn back development costs was unequivocal, *“If the patent protection is very short, they might not do it* [develop the drug]*, and then the people won't get these effective drugs … society probably shouldn't change this just like that. Surely, we are compelled to listen to the production side as well”* (I Hm2). Concomitantly, a dissatisfaction regarding the consequences of the orphan status and the market exclusivity of the drug was also found, *“… with the expenses that are charged here, you probably don't need to sell more than half a year to have those costs paid for … The orphan status is kind of starting to aggravate me a little already”* (I Ph3); *“If a drug in its first year on the market produces back its development costs by a hundredfold, it is unreasonable to through legislation protect the high price of the product with a patent for 15 years”* (M Hm2).

The fact that nusinersen is used for the treatment of a rare disease and the patient population therefore is small and nationally unevenly distributed, with some regions having dozens of patients while others have none, was an issue brought up in the discussion by several of the interview participants, *“… in a municipality, it doesn't take more than one family with three SMA-children and a few cases of difficult cancers for the said municipality's special healthcare budget to fall apart”* (I Po1); *“… it is not a good thing. That one can almost point them out, that it is that specific person who we were forced to do that for, who has cost us … no, no”* (I Po3). The uneven allocation of resident morbidity-dependent central government transfers to municipalities was also raised in this context, as regional differences in the occurrence of rare diseases negatively affect these transfers in regions with a comparatively healthy population, *“Overall, it is so, that Ostrobothnia and Åland* [provinces in Finland] *use the least of the healthcare services”* (I Pa1).

### 4.3 Demand for new strategies

#### 4.3.1 Division of responsibilities

In a relatively early stage of gathering the data, together with the ethical and financial aspects, a distinct request for clear and explicit national directives concerning both the acquisition and follow-up of orphan drugs like nusinersen was encountered. Many experts considered the financing of high-priced orphan drugs a responsible act by the government*, “… so, it is the government, but at the same time we know that the government is really tightening the belt … Therefore, it's going to be quite a difficult equation to solve”* (I Le1). As the discussion proceeded to the distribution of such drugs, the majority felt that hospital pharmacies are the best channel for distribution, *“… it feels in every way the safest, that they come to and are stored at hospital pharmacies, and are administered there. By those who know what they are doing … they are horrible sums of money. Who wants to have that as their responsibility, or store them?* (I Ph2). However, some felt that orally administered orphan drugs may well be distributed directly to the patient via community pharmacies, especially when the patient is stable and the drug is administered continuously. Interview participants from the expert groups representing pharmacies and patient organisations advocated for easier and more beneficial solutions for the patients in terms of distribution via community pharmacies in spite of the drugs being financed by public healthcare, *“… in 99 % of the cases it is easier for patients to get the drug from community pharmacies … so they could get their drugs from community pharmacies, but the financing and procurement would be handled by the hospital … Flexibility there”* (I Ph4). Some problems concerning the distribution of high-priced drugs of this kind through community pharmacies were also addressed, in terms of it currently not always being carried out in an equitable fashion concerning patients and pharmacy owners, *“… if it* [high-priced drug] *isn't reimbursed, then some hospitals have actually given the tablets to patients while some haven't … the procedure should be the same everywhere in Finland”* (I Ph4); *“… if we look at it frankly economically* [selling a drug like nusinersen from community pharmacies]*, then there is not a single pharmacy that is interested in selling this drug … Because there is not a pharmacy that would, I think, in the long run maybe make a profit from it”* (I Ph1).

The preparative work by authorities before taking nusinersen (and other orphan drugs) into use in the country was raised. In terms of the roles of involved authority agencies both positive and negative attitudes could be found, *“I think it was good that in Finland we went through quite an extensive discussion* [concerning nusinersen]*, and that we have COHERE Finland and the Finnish Medicines Agency that give recommendations”* (I Ph3); *“… how should a public health authority, the operations of which are based on the law, look at a non-binding recommendation* [by COHERE Finland] *that has such a problematic relationship with the law?”* (M Pa3). Also, the preparative work concerning price negotiations and managed entry agreements were mentioned, *“We also need to develop and improve, and actually partly even create, mechanisms according to which we then negotiate the price with the pharmaceutical company and on the whole about the entry of high-priced drugs to the market if it is decided that they will be taken into use”* (I Me2). Price negotiations with pharmaceutical companies from a payer point of view were even seen to benefit from being carried out in international forums, *“The aim would be a united front with the member countries* [EU] *with respect to the pharmaceutical companies”* (M Hm2). A centralised form of follow-up was another new strategy demand seen in the results of the study. The centralisation of knowledge on rare diseases was seen as crucial from a patient point of view in simplifying and speeding up the process of channelling patients to a proper diagnosis and care, *“The need for a centralised treatment unit is accentuated in the coming years when additional new medical treatment options enter the market and there has to be knowledge to specify what treatment is the most suitable one for which patient and if the patient needs to change the course of treatment or not”* (M Me3); *“… do maternity clinics have enough knowledge on different rare diseases? Like do they realise in time when they need to direct the patient to the special health care services?”* (I Po2).

#### 4.3.2 Political decisions

An extensive healthcare reform is currently being carried out in Finland. A concern that the reform will not provide solutions for the national issues concerning high-priced organ drugs, or even make it more complex, was clearly evident, *“In the new healthcare reform there are 18 provinces and we cannot accept a situation where the provinces end up making different decisions concerning the operational content”* (M Me1). However, some hope of a clarification to the process in connection to the reform, this at least being a possibility highly dependent on the actors in the field, was also present. Politicians being aware of difficult decision-making lying ahead with respect to high-priced treatment options was by some interviewed expert groups met with a certain concern whether politicians have enough knowledge about and insight into the matter. The sustainability of the country's social structure was another political feature found in the results of the study, addressing issues like public- contra private insurance-based systems in connection to an increasing financial polarisation between citizens, and the incremented prospects of national and international medical tourism, *“One option is medical tourism … It sounds bad, but if treatment is not received at the hospital or you cannot afford treatment on the private side, what other options are there for a patient in need?”* (M Pa4). Within the context of political decision-making concerning the issue at hand, an overall wish for openness in dialogue and shouldering responsibilities was discerned in the study results.

## 5 Discussion

This study has examined the social impact of a high-priced orphan drug, nusinersen, through the views of involved expert groups within the community. Three main themes were identified: ethical aspects; financial aspects and; call for new strategies. The results of the study show that attitudes towards nusinersen are positive overall, however, with concomitant reservations. The drug entails hopefulness, representing the first drug therapy available in treating SMA. The development of nusinersen represents in itself a major social impact for patients, families and involved healthcare professionals. However, the initial positive attitudes quickly shift to concerns regarding the escalating medical costs. Ethical and financial implications of the drug exist alongside each other regardless of the expert group showing, that different and sometimes contradictory attitudes and values compete with each other, and may be classified in different ways depending on the situation. All things considered, this leads to a call for new strategies in order to address the challenges concerning high-priced treatment options and the social impact created as a result. The social impact found within the themes and issues needing to be further addressed are discussed below.

From a patient perspective, *the need for making the voices of the patient group heard* with respect to novel treatment options like nusinersen, is recognised as a driving force for the creation of social impact. Patient organisations voice the need for communication between patients and decision-makers working with prioritisation. Decision-makers would then have a better understanding of the patients´ views of their disease and treatment options. Similar perspectives have been found in previous studies on the implications of orphan drugs.^29^, ^30^, ^31^, ^32^, ^33^, ^34^, ^35^^,^^43^ For example, in a study on differences in health technology assessment (HTA) recommendations between European countries, Nicod et al.^43^ raise the importance of the patients´ needs, preferences and values being taken into account when assessments of orphan drugs are used to inform reimbursement decisions. The importance of hearing the patient group may also be seen as recognised by COHERE Finland as the recommendations on the use of nusinersen state that the development of patient-centred indicators for assessment and follow-up are necessary.^15^ This may, together with the drug being included in the services financed by public funds, be seen as a social impact.

Patient organisations also emphasise that the drug should be reimbursed for everyone who may benefit from it and who want to try it. In addition, the overall results highlight that the treatment costs might be paid back to society if patients can contribute due to successful treatment e.g., by working. Patients being able to contribute to society to a greater degree indeed constitutes another social impact of the drug. From a patient point of view, a halted disease progression is often seen as a sufficient effect for a drug. On the other hand, many experts consider the priority setting to be fair even though it rules out a part of the patient group. They also advocate that a noticeable improvement in the health state of the patient is needed due to the high price of the drug. In a study, performed by Rouault et al. ^44^ prior to treatment options becoming available, as many as 96,5% of SMA-patients considered a halted progression as being a sufficient effect for a drug. However, since the market approval of nusinersen, the issue of the drug's benefits in comparison to the discomfort caused by the invasiveness of the treatment and other disease complications have been more recently highlighted as something that should not be overlooked as it highly affects patients´ quality of life.^29^^,^^34^^,^^35^

Finally, the patient organisations raise the fact that a Finnish patient organisation for SMA, SMA Finland,^45^ had to be founded during discussions on reimbursement in the winter of 2018 in order for the patients to be able to efficiently participate in the discussion. The founding of a disease-specific patient organisation has been previously encountered in Finland, in connection with a new high-priced treatment option for Fabry disease entering the market.^46^ This demonstrates the increased demands faced by patient organisations today, needing to be specialised within the frames of a specific disease. However, it also constitutes a social impact for patients and families, having an organisation advocating their cause alone. To summarise, the social impact of nusinersen as seen from a patient perspective is: *1) COHERE Finland including nusinersen in the services financed by public funds in Finland and acknowledging the need for patient-centred indicators for assessment and follow-up; 2) patients being able to contribute to society as a result of the effects of nusinersen; and 3) the establishment of SMA Finland as an advocate for patients and families*.

From a decision-maker perspective, *the escalating medical costs and the financial viability of the social structure* constitute the major driving force for the creation of social impact. Nusinersen serves as an example for the future regarding similar prioritisation processes and highlights the importance of sufficient reflection in the reimbursement decision. This and other studies,^23^^,^^28^ show the necessity and complexity of such efforts. They highlight limiting effects like different patient−/society concerns and call for novel frameworks for ensuring an equitable allocation of resources. Within this context the roles of involved authority agencies like COHERE Finland are also discussed. For example, the issue that the recommendations concerning nusinersen are not legally binding, but merely recommendations, is raised. Accordingly, possible regional differences in patient care due to hospitals in Finland at that time not working together with respect to the preparative work before taking high-priced drug treatments into use is seen as a problem. Since then, a national collaboration between the five catchment areas for highly specialised medical care (the national HTA coordination unit, FinCCHTA, established in the fall of 2018),^47^ has led to joint price negotiations concerning high-priced orphan drugs.^14^ This collaboration represents yet another social impact of nusinersen.

The healthcare reform that is underway in Finland, and its potential effects on the processes regarding high-priced orphan drugs, is discussed within the call for new strategies. Some experts hope that the reform will be able to clarify the processes on orphan drugs. The majority however think that the issues on the matter will not be solved as they represent such a small part in a vast entirety. The social structure of today and its sustainability in the future concerning financing of high-priced treatments with public funds is also questioned. This finding reflects the view expressed in a study by Kontoghiorghe et al.,^28^ arguing that in several countries there is a lack of both professionals and money to provide a comprehensive state-controlled healthcare service. Concerning the question of who should pay, most experts are of the opinion that the government would be the best funder for such drugs. According to them, this would provide regional justice with respect to care and allocation of resources as the current funding of high-priced treatment options is considered unsustainable. The need for and efforts to make a change are expressed in a report from 2018 by the Finnish Ministry of Social Affairs and Health on the medicines reimbursement system and may be seen as a social impact with respect to high-priced orphan drugs.^48^ However, the report suggests only ultra-orphan drugs (for ultrarare diseases affecting 1 in 50,000)^49^ to be financed on a government level. As members of the New Therapies Council in Sweden have pointed out,^50^ even though government funding would indeed reduce regional inequality, it is not a solution for using inadequate resources. Within this context, there are also reflections on the best way to distribute high-priced orphan drugs, taking into account drug safety, equality in care, and financial sustainability with respect to patients and pharmacy owners. The challenges of distributing high-priced orphan drugs via the outpatient care system is also discussed in the literature,^51^ showing that patients within the system in several European countries, like in Finland, usually pay a part of the costs themselves meanwhile via the public healthcare system they are free of charge.

The importance of follow up in patient care and more extensive collaboration forums in connection to the treatment with high-priced orphan drugs is emphasised as the number of drug treatment options are increasing, while experts in the area are few. These findings reflect the views expressed in previous research on orphan drugs, including nusinersen.^29^^,^^32^^,^^33^^,^^52^ At the time of writing, a national collaboration on rare diseases has in fact been initiated in Finland and a centre thereof has been founded in the five university hospitals of the country.^48^^,^^53^ This collaboration may also be seen as a social impact within the Finnish community. It is however important to remember that from a patient point of view there appears to be a duality within this context: while the centralisation of knowledge does improve care, it also adds burden in time, travel and other costs for patients and families. These negative consequences potentially rule out patients and add to inequalities as has been previously pointed out by King and co-workers.^29^ Taken together, the social impact of nusinersen as seen from a decision-maker perspective is: *1) the establishment of the national HTA coordination unit, FinCCHTA, and joint price negotiations concerning high-priced orphan drugs; 2) the ongoing healthcare reform and efforts to make changes to the current unsustainable reimbursement of high-priced orphan drugs; and 3) the initiation of a national collaboration and establishment of centres on rare diseases making the treatment and follow-up of patients more efficient.*

In order to create additional social impact with respect to drugs like nusinersen, issues still remaining without a solution need to be further addressed. Concerns regarding inequality within the patient group, as well as in relation to other patient groups, are found. The uncertainty regarding societal preference for rarity has been identified in other research as well.^4^^,^^43^^,^^54^, ^55^, ^56^ Further research is thus required for eliciting the social values ascribed to orphan drugs and on the implications of the prioritisation concerning relations between members within the same disease community.

Furthermore, the issue of knowledge on rare diseases on a community health centre level being too scarce in Finland, potentially delaying the most suitable treatment in the optimal time, may be argued to be a matter of medication safety.^57^ It comprises a highly relevant issue to be further addressed, as is supported by findings of a fairly recent study^34^ suggesting that more evident efficacy on functionality may be associated with an early initiation of treatment with nusinersen. The European Alliance for Newborn Screening in SMA is currently advocating all European countries to include a test for SMA in their newborn screening programmes by the year of 2025.^58^

Lastly, even though most experts understand the need for pharmaceutical companies to earn back the money invested in R&D, several criticise the unreasonable drug pricing as fairly few clinical trials are performed before approval which substantially shortens the development process. Experts point out, that as companies gain experience about a drug's effects through a controlled introduction, they should account for this in the price of the drug. They suggest that the price negotiations with pharmaceutical companies could be held in international collaboration forums e.g., between the EU-countries. As new global health policies concerning orphan drugs have also been called for in previous research,^4^^,^^28^^,^^51^^,^^53^ this strategy presents itself as beneficial in order to achieve greater negotiating power in the future.

#### 5.1.1 Strengths and limitations

The present study provides novel and detailed insight into the perceptions, thoughts and attitudes of experts on a high-priced orphan drug, thereby filling an important gap in the literature. The study has combined two different methods for data collection, namely media analysis and semi-structured qualitative interviews, to study the phenomenon. In addition, purposive and snowball sampling of interview participants from six different areas of expertise and different parts of the country, resulting in a total number of 29 media references and 16 interviews, further adds to the strength of the study and allows for a broad thematic analysis.^39^ A sufficient amount of media extracts and interview responses were also collected in order to gain saturation in the material.^40^ The interview participants had the opportunity to read and comment on the finalised results prior to publication, which further supports the credibility of the study.

The study has several potential limitations. Generalisation of the results outside of Finland may be limited as this is research heavily reliable on the healthcare system wherein it has been conducted. In addition, the study was performed during an ongoing healthcare reform. However, the results are likely to be generalisable to other Nordic countries as the healthcare systems are similar. Another limitation is due to the combination of methods as the interview guide was based on the preliminary findings of the media analysis. This may have resulted in leading questions. However, at the same time the thorough media analysis can be seen as an introduction to the subject and hence resulted in more in-depth questions in the interview guide. Also, this kind of approach has been applied in previous research.^42^ Furthermore, a pilot interview was conducted in order to test the interview guide and no need for revision was found. As the work progressed, the introductory literature search evolved into a deeper analysis of media data as experts found in media were included in the sum result. However, these experts were excluded as potential interview participants in order to broaden the foundation of the study: an increment of expert opinions from a fairly narrow pool of possibilities as the exemplifying drug is an orphan one and the study was performed in a comparatively small country. The researchers also wanted to include pharmacists in the study as the group has much information on the topic, and is underrepresented in the material from the media.

Finland has two official languages, Finnish and Swedish, which represents yet another limitation to the study. The interviews were conducted in either Finnish or Swedish and the study results were further translated into English. The first author (SR), however, is bilingual and all members of the research team have excellent skills in English.

## 6 Conclusions

The increasing occurrence of high-priced orphan drugs like nusinersen offers new treatment options to seriously ill patients but at the same time contributes to the escalating medical costs. This means that experts have to face ethically and financially difficult decision-making. However, such drugs are also found to create social impact through changes that address social injustice or challenges with respect to them. Based on the views of experts, this study shows the social impact of nusinersen thus far within the Finnish community. The social impact of the drug appears somewhat different as seen from patient and decision-maker perspectives. For patients, the most relevant social impact is the drug becoming available to at least part of the patient group, the positive effects of the drug for patients receiving treatment and the establishment of a disease-specific patient organisation advocating for them. For decision-makers, on the other hand, joint price negotiations due to the establishment of a national HTA coordination unit, the healthcare reform addressing issues with respect to high-priced orphan drugs and the establishment of centres on rare diseases. Issues that still remain to be solved and need to be further addressed include: the inequality within the patient group as nusinersen is still unavailable to a part of SMA-patients; medication safety concerns due to insufficient knowledge on rare diseases in community health centres; and the establishment of international collaboration forums for the price negotiations with pharmaceutical companies.

## Funding

This research did not receive any specific grant from funding agencies in the public, commercial, or not-for-profit sectors.

## Declaration of Competing Interest

This research did not receive any specific grant from funding agencies in the public, commercial, or not-for-profit sectors.

## Appendix A Supplementary data

Supplementary material Image 1
