# Supplementary material for: A qualitative study on the views of experts on the social impact of the high-priced orphan drug nusinersen
Source: Explor Res Clin Soc Pharm. 2023 Jan 20;9:100227. doi: 10.1016/j.rcsop.2023.100227 (PMC9918416; doi:10.1016/j.rcsop.2023.100227)
Supplement: Supplementary file 1 — Supplementary material [file mmc1.docx]

**Supplementary data**

**Appendix A.**

Information to the participant

Purpose of the study

For my master´s thesis, I have chosen to do a social pharmaceutical study on an orphan drug named Spinraza which is used for treating spinal muscular atrophy. Since the drug was granted marketing authorisation in the European Union in May 2017, the debate in Finland has been active concerning prioritisation on which patients will receive the treatment reimbursed by public funds. High-priced drugs of this kind are getting all the more common and therefore, I wish to make a qualitative study by interviewing experts within different areas who in different ways are affected by the topic, in an attempt to deepen and liaise information concerning this social consequence. The work also includes a literature study of the subject area. The interview is estimated to last for one hour at the most.

Rights of the participant

Participation in the interview is voluntary and may of the participant be stopped at any moment. The participant also has the right to leave questions unanswered. All data will be handled confidentially. No names of participants, their workplace or specific positions will appear in the study. Every person is given a code by which they are referred to. The participants have the right to gain further information on the study and will be given the opportunity to read through and comment on the work prior publication.

Use and safekeeping of interview material

The interviews will only be utilized for this specific work which will be published. The work as a whole is made in cooperation with Vaasa Central hospital. Data collection occurs by audio recording at a location chosen by the participant. The audio records will be deleted after finalisation of analysis and transcription. The material will be maintained in the main authors personal computer and therefore be protected by username.

Contact information (name, e-mail address and phone number) of main researcher and names of supervisors given at the end of the information letter.

**Appendix B.**

Interview questionnaire.

Q1. What do you know about the hereditary disorder spinal muscular atrophy (SMA) and the drug Spinraza? Have you come into contact with the illness and/or the drug before?

alternatively for experts in medicine/patient organisations:

Q1. What is your connection to the hereditary disorder SMA and the drug Spinraza?

Q2. Have you followed the discussion in media concerning the use of Spinraza in Finland (and other parts of the world)?

Q3. Initially, spontaneously and in short: What kind of thoughts does a drug of this kind give rise to in you?

Q4. What consequences do you think the prioritisation practice concerning which patients the drug is made available for through reimbursement may have? Do you think the prioritisation in Finland is fair at this point in time? What is your opinion about the prioritisation in Finland in comparison with other countries?

Q5. What do you think about the pricing of Spinraza (based on the information available)?

What is your point of view about the fact that the medical company can sell the drug to a different price to different hospitals in Finland due to the absence of national guidelines? How can this come to affect the prioritisation concerning the treatment of patients in different parts of the country?

Q6. What is your take on the operative transparency of medical companies and authorities in questions concerning orphan drugs of this kind? For example, the fact that the price negotiations between medical companies and healthcare districts are kept confidential? Do you have an opinion regarding the patent protection of new drugs?

Q7. In a near future, the global share of the total pharmaceutical market of high-priced orphan drugs is expected to rise significantly. Concomitantly, Finland is facing an extensive social- and health care reform. Ahead, who do you think should pay for drugs of this kind (municipality, landscape, health care district, state) and through which channels should they be distributed (hospital- or community pharmacy)?

Q8. Do you suppose that processes concerning this type of orphan drugs will be clarified in connection with the present health care reform or is there a risk of the situation becoming further complicated?

Q9. Is there anything else that you would like to add to the matter?

**Appendix C.**

Written informed consent for participation in interview

Name of study: Spinraza – A social pharmaceutical study

Performer of study: Sara Rosenberg, student

Supervisor of study: Jussi-Pekka Rauha, hospital pharmacist

I have been informed of the aim of the abovementioned study and of the methods used to conduct the study through an information letter to the participant. I am aware of my participation being voluntary. I am also aware of the study not costing me anything, that the material concerning myself only being used for the study at hand, that all data are handled confidentially and that the audio records will be deleted after finalisation of analysis. I give my consent to being interviewed and for the material thereby being used for the study. I may at any given moment discontinue the participation or leave questions asked of me unanswered without giving an explanation.

Place: Date:

Signature and print name of the participant

Signature and print name of study performer

Two copies have been made of this written informed consent, one of which is given to the participant and the other to the study performer.

**Appendix D.**

Table 1. Distribution of references from Finnish media used in the study.

| **Name of media / Time** | 08/  2017 | 09/  2017 | 10/  2017 | 11/  2017 | 12/  2017 | 01/  2018 | 02/  2018 | 03/  2018 | 04/  2018 | 05/  2018 | **Total** |
| --- | --- | --- | --- | --- | --- | --- | --- | --- | --- | --- | --- |
| Uly.fi | 1 |  |  |  |  |  |  |  |  |  | 1 |
| Helsingin Sanomat |  | 1 |  |  |  | 1 |  | 1 |  | 1 | 4 |
| Yle.fi |  | 2 | 1 |  | 1 |  |  |  |  | 1 | 5 |
| Lääkärilehti |  |  |  | 1 |  |  |  | 1 | 1 |  | 3 |
| Smafinland.fi |  |  |  |  | 1 | 1 | 1 |  |  |  | 3 |
| Motiivilehti |  |  |  |  | 1 |  |  |  |  |  | 1 |
| Vasabladet |  |  |  |  | 1 |  |  |  |  |  | 1 |
| Invaliidiliitto.fi |  |  |  |  |  | 1 |  |  |  |  | 1 |
| MDS-lehti |  |  |  |  |  | 1 |  |  |  |  | 1 |
| Suomen Kuvalehti |  |  |  |  |  |  | 1 |  |  |  | 1 |
| Uusi Suomi |  |  |  |  |  |  | 2 | 1 |  |  | 3 |
| Ilta-Sanomat |  |  |  |  |  |  |  | 1 |  | 1 | 2 |
| Eduskunta.fi |  |  |  |  |  |  |  | 1 |  |  | 1 |
| Turun Sanomat |  |  |  |  |  |  |  | 2 |  |  | 2 |
| **Total** | 1 | 3 | 1 | 1 | 4 | 4 | 4 | 7 | 1 | 3 | **29** |

List of references from Finnish media given in Table 1 above:

1. Uudenmaan Lihastautiyhdistys ry. SMA ja nusinerseeni Suomessa. https://uly.fi/news/sma-ja-nusinerseeni/. Accessed May 12, 2022.

2. Helsingin Sanomat. Hän on se, jonka silmät ovat täynnä iloa. <https://www.hs.fi/kotimaa/art-2000005379762.html>. Accessed November 4, 2018.

3. Yle. 11-vuotiaan pojan yksi lääkeannos maksaa 100 000 euroa – Kuka päättää, mihin yhteiskunnalla on varaa? https://yle.fi/uutiset/3-9822124. Accessed November 4, 2018.

4. Yle. Miksi lapsen lääkeannos maksaa 100 867 euroa? – Lääkemenojen kasvu kuriin salaisilla sopimuksilla uutuuslääkkeiden hinnoista. https://yle.fi/uutiset/3-9851776. Accessed November 4, 2018.

5. Yle. 11-vuotias poika odottaa Lahdessa harvinaista lääkettä, jota Turussa annetaan – "Se on epätasa-arvoista". https://yle.fi/uutiset/3-9887048. Accessed November 4, 2018.

6. Soininen M. Rajanvetäjät tarttuivat kalliisiin lääkkeisiin. *Lääkärilehti*. 2017;72(45):2570–2573.

7. Yle. Vaikeaa lihassairautta potevan 11-vuotiaan pojan äiti pettyi – yhteiskunta ei aio kustantaa kallista uutuuslääkettä. https://yle.fi/uutiset/3-9986125. Accessed November 4, 2018.

8. SMA Finland. Asiantuntijan prof Bjarne Uddin kommentit PALKOn Nusinersen-suositusluonnokseen: NUSINERSEENI SMA-TAUDIN HOIDOSSA. http://www.smafinland.fi/sman-hoito/laakkeet/spinraza/asiantuntija-prof-bjarne-uddin-kommentit/. Accessed November 4, 2018.

9. Hiilamo H. Paljonko ihmishenki maksaa? *Motiivilehti*. 2017;(10):50.

10. Furu A. Medicinen som kan hjälpa NN är för dyr. *Vasabladet*. 2017;161(302):3.

11. Helsingin Sanomat. Lääkkeen hinta ei saa määrätä lapsen tulevaisuutta. https://www.hs.fi/mielipide/art-2000005511684.html. Accessed November 4, 2018.

12. SMA Finland. SMA Finland ry:n kommentit Palkon suositusluonnokseen. http://www.smafinland.fi/sma-finland-ryn-kommentit-palkon-suositusluonnokseen/. Accessed November 3, 2018.

13. Invalidiliitto. Harvinaislääkekeskusteluun oikeudellista näkökulmaa. https://www.invalidiliitto.fi/harvinaislaakekeskusteluun-oikeudellista-nakokulmaa-1512018. Accessed November 4, 2018.

14. Auno S. Kyynelistä korvattavuuteen. *MDS-lehti*. 2018;(1):8–9.

15. SMA Finland. Heikkouksia ja rikkomuksia PALKO:n nusinerseeni-käsittelyssä. http://www.smafinland.fi/heikkouksia-ja-rikkomuksia/. Accessed November 4, 2018.

16. Soininvaara O. Tappavaa voitontavoittelua. *Suomen Kuvalehti*. 2018;102:14–15.

17. Uusi Suomi. Ylilääkäri avaa 500 000 €:n lääkehoidon taustoja: ”Kohtuutonta suojata patentilla 15 vuoden ajaksi”. <https://www.uusisuomi.fi/kotimaa/242643-ylilaakari-avaa-500-000-eun-laakehoidon-taustoja-kohtuutonta-suojata-patentilla-15>. Accessed November 4, 2018.

18. Uusi Suomi. Yksi pistos maksaa 83 000 €: Jättääkö Suomi harvinaisesta sairaudesta kärsivät lapset hoitamatta? <https://www.uusisuomi.fi/kotimaa/242797-yksi-nusinerseeni-pistos-maksaa-83-000-euroa-jattaako-suomi-harvinaisesta-sma>. Accessed November 4, 2018.

19. Helsingin Sanomat. Lääkkeen saanti riippuu nyt hintaneuvotteluista. https://www.hs.fi/kotimaa/art-2000005611534.html. Accessed November 4, 2018.

20. Lääkärilehti. Lasse Lehtonen: Lääkepatentin raukeaminen pitäisi sitoa lääkkeen tuottoon. Lääkärilehti. https://www.laakarilehti.fi/ajassa/ajankohtaista/lasse-lehtonen-laakepatentin-raukeaminen-pitaisi-sitoa-laakkeen-tuottoon/. Accessed November 4, 2018.

21. Ilta-Sanomat. NN, 8, ei saa lääkettä vaikeaan sairauteensa, koska hinta on valtion mielestä kohtuuton – isä: ”Miksi veroja maksetaan, jos hoitoa ei saa?”. https://www.is.fi/kotimaa/art-2000005592571.html. Accessed August 13, 2018.

22. Uusi Suomi. Harvinaista tautia sairastaville lapsille ehkä sittenkin lääkkeet Suomelta – jos 500 000 €:n hinta laskee. https://www.uusisuomi.fi/kotimaa/244575-harvinaisesta-sma-taudista-karsivat-lapset-saattavat-sittenkin-saada-laakkeen. Accessed November 4, 2018.

23. Eduskunta. Kirjallinen kysymys harvinaisten sairauksien hoidosta ja lääkityksestä. https://www.eduskunta.fi/FI/vaski/Kysymys/Sivut/KK_83+2018.aspx. Accessed November 4, 2018.

24. Turun Sanomat. Kuka saa jäädä ilman hoitoa? https://www.ts.fi/mielipiteet/lukijoilta/3876836/Lukijalta+Kuka+saa+jaada+ilman+hoitoa. Accessed November 4, 2018.

25. Turun Sanomat. Hukkaan mennyt investointi? https://www.ts.fi/mielipiteet/lukijoilta/3885180/Lukijalta+Hukkaan+mennyt+ininvestoin. Accessed November 3, 2018.

26. Rutanen J. Kalliista lääkkeistä on päätettävä paremmin. *Lääkärilehti*. 2018;73:1046–1047.

27. Helsingin Sanomat. Kalliin lääkkeen tekijä takoo tulosta. https://www.hs.fi/talous/art-2000005665747.html. Accessed November 4, 2018.

28. Yle. Sairaaloiden lääkekustannukset rajussa kasvussa – lääkärit joutuvat miettimään, kuka kalliita lääkkeitä saa. https://yle.fi/uutiset/3-10218186. Accessed October 31, 2018.

29. Ilta-Sanomat. Harvinaisesta sairaudesta kärsiville hyviä uutisia: Superkalliiksi arvioitua lääkettä voi pian saada Suomessa. https://www.is.fi/terveys/art-2000005664840.html. Accessed August 13, 2018.
